# Bio-Inspired 3D Infill Patterns for Additive Manufacturing and Structural Applications

**DOI:** 10.3390/ma12030499

**Published:** 2019-02-06

**Authors:** Jan Podroužek, Marco Marcon, Krešimir Ninčević, Roman Wan-Wendner

**Affiliations:** 1 Christian Doppler Laboratory LiCRoFast, Department of Civil Engineering and Natural Hazards, University of Natural Resources and Life Sciences (BOKU), 1190 Vienna, Austria; podrouzekj@gmail.com (J.P.); mmarco88@gmail.com (M.M.); kresimir.nincevic@boku.ac.at (K.N.); 2 Faculty of Civil Engineering, Brno University of Technology, 602 00 Brno, Czechia; 3 Department of Structural Engineering, Ghent University, 9052 Ghent, Belgium

**Keywords:** 3D infill, 2D infill, fused deposition modelling, digital image correlation, 3D printing

## Abstract

The aim of this paper is to introduce and characterize, both experimentally and numerically, three classes of non-traditional 3D infill patterns at three scales as an alternative to classical 2D infill patterns in the context of additive manufacturing and structural applications. The investigated 3D infill patterns are biologically inspired and include Gyroid, Schwarz D and Schwarz P. Their selection was based on their beneficial mechanical properties, such as double curvature. They are not only known from nature but also emerge from numerical topology optimization. A classical 2D hexagonal pattern has been used as a reference. The mechanical performance of 14 cylindrical specimens in compression is quantitatively related to stiffness, peak load and weight. Digital image correlation provides accurate full-field deformation measurements and insights into periodic features of the surface strain field. The associated variability, which is inherent to the production and testing process, has been evaluated for 3 identical Gyroid specimens. The nonlinear material model for the preliminary FEM analysis is based on tensile test specimens with 3 different slicing strategies. The 3D infill patterns are generally useful when the extrusion orientation cannot be aligned with the build orientation and the principal stress field, i.e., in case of generative design, such as the presented branching structure, or any complex shape and boundary condition.

## 1. Introduction

By integrating the computational design and digital fabrication process, additive manufacturing (AM) has developed over the last decades into various forms, providing architects and designers with efficient tools for rapid prototyping, individualisation and low-volume production. Conventional AM methods include stereolithography (SLA), fused-deposition modeling (FDM), and selective laser sintering (SLS), as implemented in a number of commercial 3D printing systems. As AM has become accessible, the complexity of the geometry is no longer a constraint, as will be shown in this paper, where the layer-based FDM method is utilized.

Although 3D printing is traditionally utilized for rapid prototyping and for high-value, low-volume production, such as in the aerospace industry, the last years have seen many successful efforts in upscaling the 3D printing systems to structural and architectural scales. Most of such large scale 3D printing systems are, however, still to be considered rather experimental. This also applies to autonomous construction systems in general, where autonomous and self-sufficient robots perform not only the 3D printing, but also manipulation or excavation tasks [1].

In comparison to the proposed autonomous construction systems, the traditional fabrication technology in civil engineering can be considered slow, labour intensive, dangerous, expensive, and constrained to primarily rectilinear forms, often resulting in homogenous structures. The associated number of annual fatalities is estimated to 50,000, which amounts to 17% of all workplace accidents.

The shift to autonomous construction systems could potentially improve safety, speed, and quality. Furthermore, this technology enables the operation in inhospitable and extreme environments, such as arctic environments or those caused by natural disasters. The extra-terrestrial application of autonomous and self-sufficient 3D printing is considered to be the primary construction technique involved in the colonization of Mars [2,3]. It is also believed, that autonomous construction systems could easily adapt to site-specific conditions and constraints and, thus, contribute to sustainable construction in general.

By accepting 3D printing as part of the future of civil engineering and construction, the multiscale optimization of structures, i.e., the outer shape of the structure and the inner configuration of materials (or infills), becomes quite desirable as the geometrical complexity of both no longer represents an obstacle. For example, some of the presented structural patterns (3D infills) cannot be manufactured by conventional approaches, such as extrusion or moulding, with the exception of weaving. However, the latter is limited to several periodic minimal surfaces and scales beyond practical use in structural engineering.

The 1st RILEM International Conference on Concrete and Digital Fabrication (2018) is another indicator showing the growing interest of the concrete community in AM with cementitious materials.

Possible applications in the near future may include on-site 3D printing of entire structures or structural members, or prefabrication of structural members or small structures. In terms of successful applications of large scale 3D printing or robotic fabrications, new experimental realizations emerge more and more often both from academia and industry, such as the contributions from IAAC (see e.g., [4]), the autonomous construction rig from MIT [1] or the 100 square meter house 3D printed in cement for the Milan Design Week 2018 (CLS Architetti), to name the very few.

From a structural point of view an interesting alternative to the layer-based 3D printing is the stress line additive manufacturing (SLAM), which has been proposed recently by [5]. The deposition of filaments along the principal stress lines improves the anisotropic limitation of the conventional layer-based FDM, but may require more forming material (supports).

Layer based 3D printing at architectural scales with cementitious materials or straw reinforced mud is implicitly considered in the following discussions and motivations, while the experimental campaign and mechanical characterization is based on small-scale layer-based FMD using PLA (polylactic acid). Note that the current market with 3D printing filaments offers stronger materials than PLA, such as Polycarbonate (40% higher tensile strength). However, the purpose of this study was not an optimization of load carrying capacity but instead to investigate the potential of different infill patterns. The authors believe that some of the principles and results stemming from the small scale campaign will be generally valid and valuable for the consequent design of real-scale experiments with structural materials, such as concrete.

After introducing the background of topological optimization and the infill options considering the technological constraints of 3D printing, an experimental campaign aiming at a quantification of infill options in terms of load capacity/weight ratio and stiffness/weight ratio is presented. Finally, the results are generalized utilizing elastic finite element analysis. All presented figures are based on results generated by the authors.

### 1.1. Motivation by Topology Optimization

Although the classical theories of continuum and discrete mechanics can approximate the stress field reasonably well for any given structural member, the final design of such member rarely reflects the principles of optimized inner forces given a certain material, as many fabrication constraints have to be taken into account. Examples are the cost of the formwork or concrete casting and consolidation [6].

If such constraints are removed by the 3D printing process, existing numerical methods can be used to optimize the material utilization within a given structure with respect to given loading, boundary conditions and other performance oriented goals (e.g., acoustic or thermal). Not surprisingly, such optimization results in lightweight, non-prismatic and streamlined structures, often resembling shapes which can be found in nature at various scales—from branching structures like trees down to biological membranes. Please note that the term bio-inspired or organic is often used by architects and designers who apply or get inspired by the principles from nature, while, the same design principles can emerge from a simple numerical optimization based on straight forward mechanical principles. Through this paper the term organic refers to the outer shape of a structure, directly obtained by numerical topology optimization. The term bio-inspired, furthermore, refers to the infill patterns, the shapes of which do not originate from a topology optimization process but were described by mathematicians who supposedly were inspired by nature some 140 years ago.

**Figure 1:**
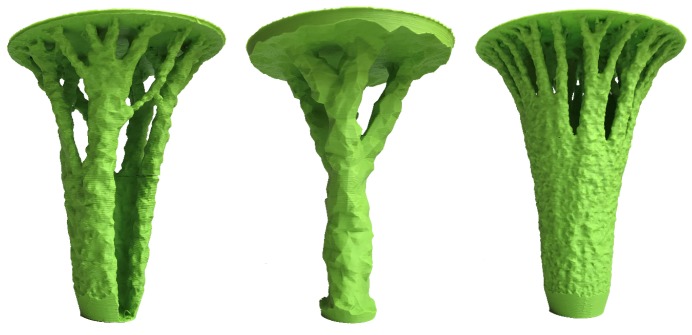
Transition from columns to branching structures: 3D printed samples from FEM topology optimization, the finite elements were intentionally unaltered to show the effect of removal of elements and the effect of element size on the overall shape.

**Figure 2:**
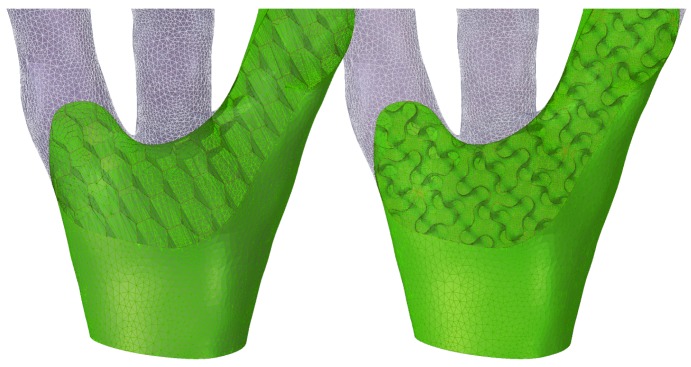
Application of classical 2D (hexagonal, left) and non-traditional 3D infill (gyroid, right) to a detail from the branching structure from Figure 1 (middle): the 2D infills are sensitive to orientation and therefore not suitable for complex 3D geometry.

A transition from classical column to (organic) branching structures is shown in Figure 1, for which a linear Finite Element Model (FEM) model was repetitively run while removing the least-utilized material with respect to stress. This example can be interpreted as roof support, and serves as a motivation for the subsequent analysis of various infill options. In 3D printing rarely infill densities close to 100% are used. Figure 2 shows a detail of this branching structure (15% infill density), which illustrates how classical 2D infill patterns (such as honeycomb) cannot be aligned with the principal stress direction, while the gyroid 3D infill forms a porous infill with constant curvature, no planes of symmetry and no embedded straight lines. Such infill properties without preferential direction are useful when complex 3D geometries under general stress states are considered.

**Figure 3:**
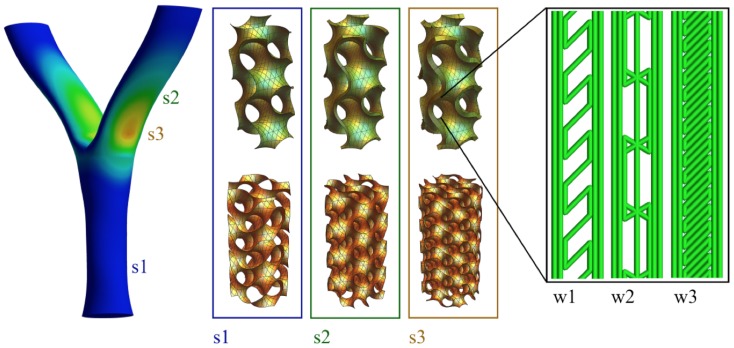
Principle of multiscale optimization utilizing a regular gyroid lattice mapped to stress levels (s1 to s3) by manipulating the 3D infill wall thickness or scale of the 3D infill. The wall density can further be adjusted by varying the wall infill strategy (w1 to w3).

3D printing also opens the door to optimizing the local infill density based on the actual stress fields and performance requirements, i.e., to manipulate infill wall thickness or infill feature size in order to achieve uniformly distributed stress (Figure 3). As this mapping can be fully automated and integrated in the computational design and digital fabrication process at no additional cost, there is no reason not to consider it.

### 1.2. Infill Patterns

**Figure 4:**
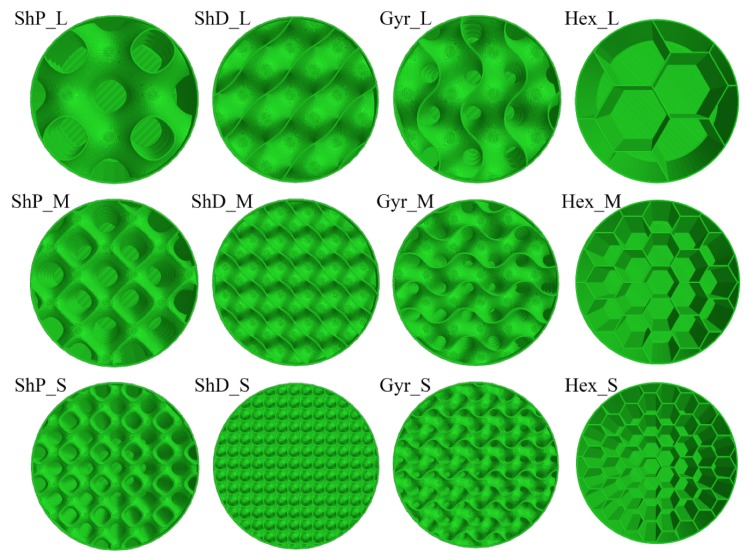
Overview of tested 3D and 2D infill alternatives (left to right): Schwarz P, Schwarz D, Gyroid and Hexagonal; and sizes (top to bottom): Large, Medium and Small.

In 3D printing, the structure that is printed inside an object is referred to as infill. It is generated in a designated pattern and percentage, which is governed by the selected thickness and scale. The infill settings are typically handled by the slicing software and may influence the material usage (weight), strength and print time. Alternatively, the infill patterns may be directly modeled by any general purpose CAD (computer aided design) or computer algebraic system. The latter approach was chosen for this investigation of the presented 3D infills (honeycomb is not considered 3D infill), which are not currently available in any slicing software, according to the authors’ knowledge. The presented examples and printed test specimens were generated by using a computer algebraic system and tested by the engineering simulation software ANSYS [7]. Generally, there are two options on how to generate 3D infills. The first is to apply parametric equations approximating the shape while the second is to copy, rotate and mirror basic building elements. The conventional 2D infill patterns include rectilinear, grid, triangular, wiggle and hexagonal (selected, Figure 4).

The 3D patterns were selected from a family of triply periodic minimal surfaces (TPMS) [8,9], which are known to be invariant under a rank-3 lattice of translations and for having a zero mean curvature (a surface that locally minimizes its area). In the real world, examples of TPMS include some biological structures in nature, block copolymers and electrostatic equipotential surfaces in crystals. According to [10], most TPMS forms exist as an interface between two phases. In particular, a gyroid, Schwarz D and Schwarz P were selected (Figure 4).

The gyroid was first described by NASA scientist A. Shoen in 1970 [9]. In nature, self-assembled gyroid structures can be found in certain surfactant or lipid mesophases or block copolymers [11], inside cells [12] or in biological structural coloration [13], to name the few. The mechanics of a pristine gyroid graphene structure have been recently investigated by [14], who, with respect to engineering applications, pointed out its ultralight nature, outstanding mechanical properties, high surface area, and stable chemical and thermal properties.

The gyroid surface can be trigonometrically approximated by the following equation (implicit surface):

(1) sinxcosy+sinycosz+sinzcosx=0

The Schwarz D and P surfaces were first described by H. A. Schwarz and his student E. R. Neovius in the 1880s [15]. Both the D and P surfaces have been considered for prototyping tissue scaffolds with a high surface-to-volume ratio and porosity or compact light-weight fuel cells with high energy density [10,16].

The Schwarz D surface can be approximated by the following equation:

(2) sinxsinysinz+sinxcosycosz+cosxsinycosz+cosxcosysinz=0

and the Schwartz P surface can be expressed as:

(3) cosxcosycosz=0

The investigations of tailored materials are typically carried out at lower scales, where hierarchical structures are studied due to their exclusive properties, such as ultralight nature, high surface are-to-volume ratio or chemical reaction efficiency. Various applications include drug-eluting devices, water purification filters, or energy-harvesting devices. At the nanoscale, e.g., a 3D printed gyroid with embedded photocatalytic ability was recently described by [17].

## 2. Experimental Investigation

**Figure 5:**
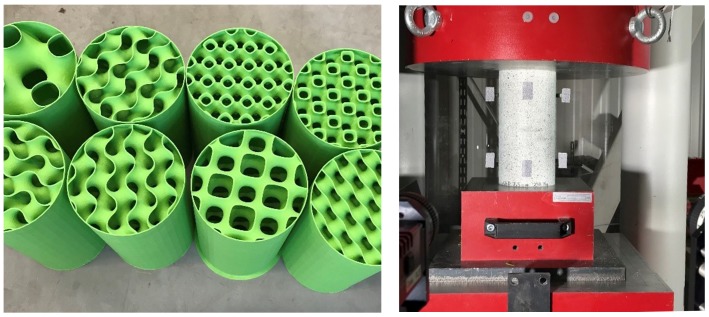
Particular batch of 3D printed specimens with various 3D infill types and sizes (**left**), and cylinder compression test setup in hydraulic press with a stereoscopic digital image correlation system (**right**).

The purpose of the experimental investigation was to analyze the qualitative and quantitative differences in mechanical response between the investigated 3D infill patterns (3 types) in comparison to the reference 2D pattern (honeycomb). This data will serve for the validation of computational models in order to investigate also more complex stress states. By considering 3 length scales per each infill type, 12 combinations of infill patterns have been generated inside a cylinder (Figure 4) and 3D printed (Figure 5, left). In case of one gyroid geometry three specimens were printed in order to characterize the inherent production and testing process variability. In total 15 cylinder specimens were tested in a standard compression test configuration (Figure 5, right).

The common properties of all tested cylinders are height (200 mm), diameter (100 mm), constant wall thickness of two perimeters (2 × 0.45 mm extrusion width = 0.9 mm). Unfortunately, the pure infill pattern can not be printed due to limitations in the production process. A bottom solid face is necessary as a stabilizing base, avoiding the otherwise necessary brim, i.e., additional perimeters extending outward from the object. Similarly, the cylindrical jacket is required in order to avoid supports. In order to quantify the potentially confining effect of these required elements extra specimens have been tested (i) without the outside walls; (ii) without bottom face; and (iii) with 0% infill (just cylinder jacket).

**Figure 6:**
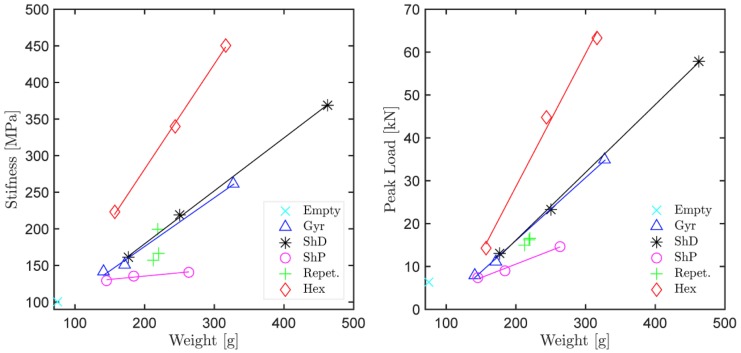
Mechanical performance of tested lattices as a function of weight: stiffness and peak load capacity.

**Figure 7:**
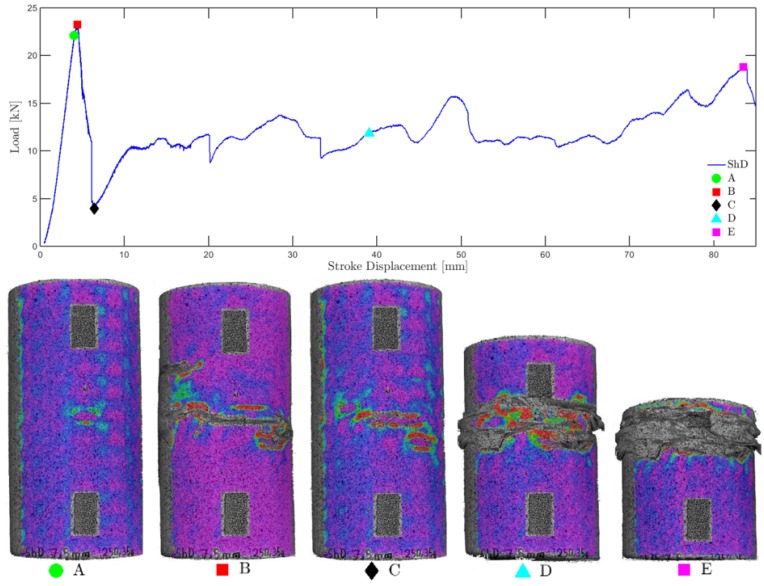
ShD_M: illustration of sequential collapse using Load-Displacement diagram and damage localisation based on 5 DIC strain fields (Lagrange, horizontal component, A to E) mapped to black and white photos of the compressed specimens.

The mechanical characterization of the test specimens was carried out by a hydraulic compression testing machine in combination with a 3D digital image correlation (DIC) system (Figure 5, right), enabling the measurement of full strain and displacement fields based on which accurate estimates of the macroscopic stiffness and load-displacement diagrams were obtained. (Figure 6 and Figure 7). Each compression test was controlled by a prescribed displacement rate with a total displacement limit of 85 mm (0.42 strain).

**Figure 8:**
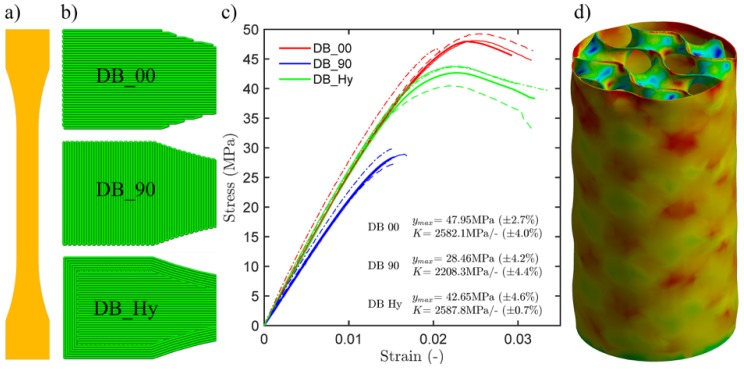
Material characterization (based on ISO tensile test specimens, (**a**) and 3 different slicing strategies, (**b**) for nonlinear FEM analysis (**c**) and resulting periodic strain field (**d**).

The tensile characterization of the PLA filament was performed using classical ISO (see [18]) dog bone test specimens, which were sliced according to 3 different strategies, varying infill angle and number of perimeters (Figure 8a,b, all 100% infill).

All test specimens, i.e., cylinder and dog bone specimens, were printed by the layer-based FDM method from the same batch of PLA filament in order to minimize systematic bias due to production and storage. A serially-produced printer model Prusa i3 MK2 (Prusa Research, Prague, Czechia) with a 0.4 mm nozzle was used together with the slicing software Simplify3D (4.1.1, Simplify3D, LLC., Cincinnati, OH, USA), as the infills were modeled externally and not generated by the slicing software. The following parameters were considered:

- layer height: 0.15 mm,
- outline/perimeter shells: 2 (×0.45 mm), including the infill,
- build plate temperature: 55 ${}^{{}^{\circ}}$C,
- printing head temperature: 215 ${}^{{}^{\circ}}$C,
- default printing speed: 2400 mm/min.

Random start points for all perimeters were used in order to avoid a seam which could affect the mechanical properties. This resulted in longer printing time due to significant increases in travel moves. The printing time averaged around 30 h per specimen and the weight averaged around 150 g per specimen.

## 3. Results

The results of the cylinder compression tests are presented and discussed in subsection *Experimental* and relate to the performance of various infills (metamaterials, 3D materials), while the results of the dog bone tensile tests are presented in the subsection *Numerical*, where they relate to the actual PLA material and anisotropy of 100% infill resulting from different slicing strategy. The latter is used to calibrate the nonlinear PLA material model for the preliminary finite element modelling framework, which is validated by the former. The numerical model will consequently serve for the design of component-scale experiments, such as the presented branching structure (Figure 1 and Figure 2), and as a proof of concept is presented here with limited details.

### 3.1. Experimental

The performance of the tested 2D and 3D infills at various scales (densities or weights) has been quantitatively evaluated in terms of stiffness and peak load in Figure 6. Here, various functional forms have been considered for the scaling laws, however, linear interpolation seemed most appropriate. The linear trend lines show that both in terms of stiffness and peak load, the 2D hexagonal infill outperformed the tested 3D infills, which is not surprising considering that the extrusion axis of the pattern is aligned with the axis of the macroscopic member (cylinder) and the principal stress field. Note that such conditions are very specific and generally not possible due to geometrical reasons and the multitude of possible loading scenarios in real world applications, such as presented in the example of a branching structure (Figure 1 and Figure 2). Among the investigated 3D infills the Schwarz P surface is the least performant infill type, while the Schwarz D and Gyroid perform similarly well.

The associated variability, which is inherent to the production and testing process, has been characterized based on three identical Gyroid specimens (Figure 6, green crosses “Repet”) in Table 1. For a reference, the confinement itself (specimen with 0% infill, Figure 6, turquoise x mark “Empty”) has been tested, together with specimens without confinement walls (difficult to print without supports) and with confinement walls but without the bottom face (0.54% difference).

The recorded load-displacement diagrams and strain fields from DIC finally serve for analyzing qualitatively differences in ductility, strain periodicity resulting from the hierarchical lattice, and a sequential collapse with damage localization (Figure 7). Although all investigated specimens exhibited large deformation capacity with strains of up to 0.42 owing to the infill patterns and the chosen PLA filaments, the specimens with 3D infills exhibited smoother strain fields in general, especially compared to the 2D hexagonal infills. This effect can be attributed to local buckling and stress concentrations which are significantly reduced by 3D infills (shorter buckling length) and can also be observed in the computational simulation results (Figure 8). The sensitivity of the layer-based FDM process to slicing strategy and build orientation based on tensile dog bone specimens is statistically evaluated in Figure 8c. Finally, that data serves as input for the calibration of nonlinear material models.

### 3.2. Numerical

The classical cylinder compression test results show, among others, that when a 2D infill pattern is aligned with the principal stress orientation, the 2D infill may outperform the 3D infill. The general design, however, typically involves more complex shapes and boundary conditions, which cannot be easily approximated by classical mechanical tests, and where there is no preferable extrusion orientation for 2D infills. In such cases, 3D infills may represent more efficient alternatives to classical 2D infills, if properly understood and designed. This clearly cannot be done without a computational model (e.g., FEM) calibrated and validated by data from classical physical experiments. These should entail compression and fracture tests, and account for any anisotropy due to printing artifacts.

In a first approach we characterize the rather complex anisotropic behavior associated with layer-based production processes by simple tensile tests on standard “dog bone” (DB) specimens (Figure 8a). As the slicing strategy and build orientation may significantly affect the mechanical performance, the DB specimens have been sliced in 3 ways (Figure 8b) according to [18] with three repetitions for each scenario to generate a minimum of statistical insights. The results are shown in Figure 8c. Here, the slicing strategy DB_00 refers to the case where the extrusion direction was aligned with the loading direction (no perimeters used), providing maximum load capacity, DB_90 is the most ineffective strategy, where the extrusion direction is normal to the loading direction and no perimeters are used. DB_Hy refers to a more traditional slicing strategy, where 6 perimeters are used and the remaining volume is sliced as a DB_00 and DB_90 repetitive sequence, i.e., each infill layer has an infill angle normal to that of neighboring layers. At this point further material tests characterizing e.g., the interface properties between layers in mode I and mode II have not been performed.

The stress-strain relationships from the ISO tensile tests (DB) have been used to formulate a custom nonlinear material model for PLA for preliminary static structural analysis (ANSYS${}^{®}$ Mechanical™) in order to simulate the physical experiments. The number of nodes amounts to approx. $1.2\times 10^{6}$ and the elapsed run-time amounts to approx. 50 h at a standard workstation computer with 4 cores operating at 4 GHz with 16 GB RAM. With the exception of the material properties all other solution parameters were automatically set within mechanical physics preference.

The numerical results coincide with the experimental data by approx. ±10%. An example of a simulated cylinder with gyroid medium infill (Gyr_M) can be seen in Figure 8d). The external surface exhibits periodic features in the strain field, similar to those observed by digital image correlation in the actual experiments. These are unique to the gyroid infill type. Unlike the DIC results that are limited to the surface the numerical analysis provide interesting insights into the sequential collapse mechanism by allowing to trace also the porous inner structure hidden to the optical DIC system.

In a next step the computational model may further be used to explore more efficiently the high-dimensional design space with respect to different geometries, lattice types, scales, and associated parameters.

## 4. Discussion

It is well known that the structural performance of layer-based FDM printouts can be significantly anisotropic (sensitive to build orientation). By properly aligning the filament deposition orientation and structural action, the structural performance may increase by up to 60%.

This may be sufficient for simply supported and loaded components, such as the tested cylinder mimicking a column under centric compression. However, for complex streamlined structural components such as the presented branching structures (Figure 1), the (organic) shape of which is often governed by optimization (generative design), it is not possible anymore to align the infill orientation with the direction of maximum principal stresses.

Therefore, a variety of (bio-inspired) 3D infills have been proposed and analyzed as a layer-based FDM-compatible alternative to currently used 2D infills. Note that the presented 3D infills can only be (continuously) fabricated by 3D printing (at the presented scales). Thus, any attempt to actually characterize their mechanical performance had to wait for more than hundred years for the AM to fully develop.

Among the most structurally important aspect of the presented 3D infills is the double curvature which provides the required stability (stiffness). Moreover, the triply periodic minimal surface of the gyroid (3D infill), known for its constant curvature, has no planes of symmetry and no embedded straight lines, which is useful when filling the above-mentioned complex geometry regions.

The 2D infills, such as rectangular or hexagonal grid, are not generally suitable for structural applications with complex 3D geometry due to the resulting anisotropy (sensitivity to the orientation of the 2D lattice). However, in case of simply loaded and supported classical linear structural elements 2D infills may outperform 3D infills.

If 3D printing is the future of construction in civil engineering, then the structural members no longer have to be limited to rectilinear and solid bodies. In these cases a multi-scale approach in the design optimization, which will lead in some cases to organic streamlined structures with bio-inspired infill patterns, will bring many new opportunities. These will be trailed by challenges, as the current design guidelines, safety concepts, and performance indicators will have to be thoroughly reviewed and possibly adapted for AM.

## 5. Conclusions

The particular results of the mechanical characterization of the 2D and 3D infills can be summarized as following:

- Stiffness (modulus) results obtained by digital image correlation (DIC) show a linear scaling law with infill density, COV of 12.92% and equally performant Gyroid and Schwarz D.
- Load capacity (peak stress) results show a linear scaling law, COV of 5.92% and equally performant Gyroid and Schwarz D.
- All investigated specimens exhibit ductile behavior (from load-displacement diagrams) and the specimens with 3D infills smooth strain periodicity without stress concentrations (when compared to 2D hexagonal infills).
- The sensitivity to slicing strategy and build orientation was evaluated on a dog bone tensile test specimens, resulting in up to 60% difference and relatively small scattering (COV ranging from 0.7 to 4.6%).

The preliminary nonlinear FEM analysis calibrated by the tensile dog bone specimens shows a good qualitative agreement with the experimental results in terms of peak load, stiffness and periodic strain fields. However, the calculations are extremely computationally expensive making any systematic upscaling to large structures an unfeasible proposition.

Based on the presented theoretical arguments and experiments it can be assumed that custom 3D infill patterns may be an interesting alternative to classical (2D) infills in additive manufacturing if (i) general stress states are likely to occur; or (ii) in case of complex geometries. 3D infills avoid introducing macroscopic anisotropy by additive manufacturing while still achieving large weight reductions.

Lightweight generative multi-scale design can be applied at architectural scales (Figure 1), potentially offering additional benefits in terms of tuned thermal, acoustic, static or dynamic properties. This would, however, require a highly scalable simulation framework to support the representation and simulation of material structure and evolution across multiple length and time scales, such as the Digital Material concept.

## Figures and Tables

**Table 1:** Production and testing process variability based on 3 identical Gyroid specimens (* Coefficient of variation).

	Weight	Peak Load	Stiffness
Mean	217.47 g	15.84 kN	174.43
COV *	1.87%	5.29%	12.92%
